# Farmer-suicide in India: debating the role of biotechnology

**DOI:** 10.1186/s40504-017-0052-z

**Published:** 2017-05-11

**Authors:** Gigesh Thomas, Johan De Tavernier

**Affiliations:** Faculty of Theology and Religious Studies, KU Leuven, Sint-Michielsstraat, 3000 Leuven, Belgium

**Keywords:** Farmer-suicide, Monsanto, Navdanya, Biotech, Bt. cotton, Vandana Shiva, Ethics

## Abstract

Indian Biotech opponents have attributed the increase of suicides to the monopolization of GM seeds, centering on patent control, application of terminator technology, marketing strategy, and increased production costs. The contentions of the biotech opponents, however, have been criticized for a lack of transparency in their modus operandi i.e. the use of methodology in their argumentation. The fact is, however, that with the intention of getting the attention of those capable of determining the future of GM cotton in India, opponents resorted to generating controversies. Therefore, this article will review and evaluate the multifaceted contentions of both opponents and defenders. Although the association between seed monopolization and farmer-suicide is debatable, we will show that there is a link between the economic factors associated with Bt. cultivation and farmer suicide. The underlying thesis of biotech opponents becomes all the more significant when analysed vis-à-vis the contention of the globalization critics that there has been a political and economic marginalization of the Indian farmers. Their accusation assumes significance in the context of a fragile democracy like India where market forces are accorded precedence over farmers’ needs until election time.

## Introduction

India has witnessed around 300,000 farmer-suicides over the past two decades (Philpott, 2015; Mishra, 2014). Biotech opponents attribute the majority of these suicides to the monopolization of the cotton seed sector. Suspected links surfaced 3 years before the seed’s commercialization by Monsanto (Shiva, 2011b; 2013a; Shiva et al. 1999; Stone, 2010a; 2011b; 2013c; 2013d; 2014b; Jahnbegloo, 2013). They characterize this distressing phenomenon as ‘genocide’, averring that such suicides were unheard of prior to the commodification. These opponents argue that the 2002 introduction of Bt. cotton dramatized the situation (Navdanya, 2012; Shiva and Jalees, 2006; Shiva, 2013b). Holding transgenic seeds responsible, they dubbed them “Seeds of Suicide, Seeds of Slavery, and Seeds of Despair” (Shiva and Jalees, 2006, p. 281; Shiva, 2013c). Huge media coverage and a parliamentary committee report have intensified the debate. The *Acharya* report attributes the increase of farmer-suicides after 2002 to Bt. cotton cultivation (Ministry of Agriculture, 2012; Sainath, 2012; Malone, 2008). Similarly, *Daily Mail* and *The Guardian journalists* and the award-winning documentary *Bitter Seeds* also link Bt. cotton cultivation with farmer-suicide. This linkage was alleged also by Charles, the prince of Wales (Malone, 2008; Faife, 2012; Lean, 2008). These allegations have been criticized by others for their lack of scientific rigor, especially the lack of transparency in their methodology (Herring, 2006; 2008; 2014; Stone, 2011b).

Therefore, we intend to critically evaluate the pros and cons, focusing on the veracity of the alleged link between GM cotton cultivation and farmer-suicide. Before we start doing this, we describe a succinct history of GM cotton and the opposition against it, and evaluate the significance of the contentions of the biotech opponents by positioning their criticism within the larger debate on the deleterious impact of economic liberalization on Indian farmers.

## A succinct chronology of Bt. cotton in India

In 1990, Monsanto requested authorities to conduct field trials. The request was rejected in 1993 by India’s Department of Biotechnology (DBT), the authority for monitoring elementary and small-scale tests, because of the exorbitant trait fees and presumed problems crossing an American variety with an indigenous one (Gupta and Chandak, 2005). Instead, the DBT preferred that Bt. genes be directly incorporated into the indigenous variety. However, in 1995, Mahyco was permitted to conduct field trials with local varieties backcrossed with Monsanto’s imported 100 g gene. Soon, Monsanto participated directly in the experiment by buying a share in Mahyco in 1998 resulting in Mahyco-Monsanto Biotech (MMB) (Gupta and Chandak, 2005; Newell 2007). In June-July 1998, before securing the DBT’s permission, MMB initiated field trials in 40 one-acre-plots in Andhra Pradesh, Punjab, Haryana, Maharashtra and Karnataka. The Genetic Engineering Approval Committee (GEAC) of the Department of Environment, Forest and Wild Life (DOE) - India’s highest authority on large-scale experimentation and commercialization – was not satisfied with the DBT’s observations on bio-safety because the trial was allegedly conducted during times of low pest presence; therefore, on 19 June 2001, it ordered a repetition of the field trials. The GEAC field trials were to be monitored by the Indian Council of Agricultural Research (ICAR) (Cohen and Paarlberg, 2004; Jayaraman 2000; 2001; Raghuram 2002). On 26 March 2002, the GEAC conditionally approved the commercialization of four Monsanto hybrids: MON 531 (bollgard 1), MECH 12, MECH 162 and MECH 184 - for a 3 year period from April 2002 - March 2005 (Qaim et al. 2006; Raghuram, 2002; Jayaraman and Bouchie, 2004; Jayaraman, 2002; Stone, 2007c; Herring, 2015). Because of the perceived fear of the seeds’ susceptibility to leaf curl, the Committee declined permission to commercialize these seeds in North India. Subsequently, the GEAC approved one more hybrid in 2004 and 16 more in 2006 using event MON 531 and event 15985 for MMB (Gruère et al. 2008).

Another facet of the chronology was Monsanto’s shrewd business move vis-à-vis accusations of preferential treatment by the regulatory authorities. Monsanto’s alliance with Mahyco was a calculated business gambit as Mahyco’s director Dr. Badrinarayan Barwale, who, as the 1998 recipient of World Food Prize, enjoyed a good reputation in government circles and had a sound rapport with many officials of the key biosafety regulation agencies (Scoones, 2003; Newell, 2007). This “proprinquinity” between Mahyco and the regulatory agencies engendered skepticism about the effectiveness of GEAC’s monitoring of MMB’s field trials (Scoones, 2003) - a good example of *Quis Custodiet Ipsos Custodes*? There were accusations of collusion by overlooking regulatory procedures by GEAC’s scientific advisor P.K. Ghosh. Consequently, opponents like Shiva and others introduced a lawsuit in India’s apex court questioning DBT’s authority to grant approvals for the trials (Newell, 2007).

A third aspect consists of allegations leveled against MMB's GM cotton, such as causing livestock death due to usage of the restricted patented gene (GURT) - termed the “terminator” by the Canadian NGO Rural Advancement Foundation International (RAFI) (Herring, 2009; 2010; 2012). In March 1998, RAFI uncovered the development of this terminator technology by the American firm Delta and Pine Company with the financial assistance from United States Department of Agriculture (USDA). As soon as this information became known in India, it triggered a public outrage. Incidentally, this public outrage coincided with MMB‘s field trials, and the failure of that cotton crop aggravated the public's fear of allowing terminator technology in India. The opposition started with public protests which culminated in the burning of the Bt. trial fields in Andhra Pradesh and Karnataka in November 1998 (Bharathan, 2002).

The fourth aspect is the fast-paced adoption of Bt. cotton in India. As of 2011/2, i.e., less than a decade after its legal commercialization, GM cotton commanded an 88% share of the total cropped area of cotton (from 0.05 million ha in the first season to 9.3 million ha in 2012) (Kolady and Herring, 2014).

Finally, it is important to note that not all GM cotton seeds are MMB cotton seeds. As of 2009 there were 6 other legal GM brands available: 2 more by MMB, and one each by Nath Seeds, JK Agri-Genetics, Metahelix Life Sciences, and Central Institute of Cotton Research along with the University of Agricultural Sciences, Dharwad (Karnataka). What is more, unofficial transgenic seeds are also in use. In 2001, the GEAC banned the Navbharat 151 when it discovered that the seed contained the illegally incorporated gene of Monsanto (Herring, 2013; 2015). Although there are no precise data on the use of illegal transgenic seeds, a 2012 study suggests that illegal GM cotton was cultivated on 1.2 million ha vs. legal seed on 6.2 million ha by 2007 (Herring, 2013; Ramaswamy et al. 2012a, 2012b). The use of non-MMB illegal seeds notwithstanding, Monsanto has borne the brunt of the controversies (Herring, 2008).

## The campaign against gm cotton

No sooner had Monsanto imported the Bt. cotton gene in 1998 than the indigenous NGOs and activists began the struggle against it. The *Karnataka Rajya Ryota Sangha* (KRRS - the erstwhile Karnataka farmers’ movement) declared the ‘Cremate Monsanto’ movement. Sangha warned that all trial fields in Karnataka would be set ablaze with the media present. The farmers of Bellary and Raichur districts consented to the arson. The crop-burning was repeated in 2002 following the official release of GM seeds. This protest had a similar outcome; some farmers received compensation from the KRRS and others sought police protection (Scoones, 2008). Apart from this media drama, the KRRS also demanded a 5-years ban on GM cotton seeds (Scoones, 2005).

The longest opposition came from Vandana Shiva and her associations, Research Foundation for Science and Technology (RFSTE) and Navdanya, through activist writings, public interest litigations (PIL) and agitation-events, e.g., seed tribunals attended by farmers from Europe, the U.S. and India (Scoones, 2003). Her advocacy began immediately with a PIL in the Supreme Court on the integrity of the genetic engineering regulation procedures. She contended that Review Committee on Genetic Manipulation’s (RCGM) permitting the field trial by Mahyco-Monsanto violated the 1989 Amendment of the Biosafety Rules that had vested the GEAC and not the RCGM with the authority to permit trials. Ever since Shiva’s PIL, courts were inundated with similar cases filed under the Freedom of Information Act, like the petition to release trial results in Delhi alleging the Gujarat government’s connivance with Mahyco-Monsanto, and the petition in Mumbai demanding compensation for crop loss under the Plant Variety and Protection Act by farmers’ movements in Vidarbha (Scoones, 2003). The Court responded to Shiva’s petition by ordering a temporary ban on field trials until the GEAC could guarantee the non-endangerment of human health and the environment. The DBT, in turn, amended the law and empowered the RCGM to grant multi-locational small-scale trials for a total of 20 acres, of up to 1 acre each (Damodaran, 2005). This court case and the alleged use of the terminator technology, discussed below, enabled farmers to cautiously adopt the technology (Newell, 2008).

In 1999, GM opponents contended that illegal field trials had been on-going since June 1998 (Shiva et al. 1999). The significant outcome of this controversy was the GEAC’s refusal of Mahyco’s 2002 appeal for commercialization of the transgenic cotton (Iyengar & Lalitha, 2002). In 1999, when Monsanto advertised the benefits of GM cotton, Shiva’s Navdanya and other anti-GM organizations launched the Monsanto Quit India Movement to raise public awareness on the negativities of GM crops. Every August 9th, Navdanya continues this campaign [http://navdanya.org/campaigns/seed-sovereignity; Scoones, 2003). The acme of opposition was the combat against MMB’s plan to employ the terminator technology. Because of the protest by Shiva and others, the Indian government banned this technology on 25 May 1998. Although Monsanto intended to commercialize the technology, Monsanto promised in 1999 to not commercialize it due to the agitation (Glover, 2007a; 2007b; 2010b; Shah, 2005; Herring, 2007b; Stone, 2002b; Monsanto, 2000).

The *Bija Panchayat* (Seed Tribunal), launched in 2000, is another venue used by GM opponents to attack GM cotton (Navdanya, 2012). The format is a kind of trial of a carefully or haphazardly chosen representative group of the general public by a jury involving a formal process. The jury generally condemns the GM cotton. These tribunals gathered national and international media attention (Scoones, 2005).

## The seed monopolisation debate

Shiva asserts the driving force behind farmer-suicide swirls around Monsanto’s monopolization of the cotton seed sector by patent control, removing alternatives, the increase in production costs, and the fear for crop failure. On account of these allegations, MMB’s Bt. cotton was banned in August 2012 in Maharashtra (Press Trust of India, 2012), but was later lifted in May 2013 (Deshpande, 2013).

### Farmers’ free saving/exchange of seeds vs MMB’s patent control

Opponents accuse MMB of exploiting intellectual property and patent laws to gain suzerainty over the cotton seed sector. In *Protect or Plunder*, Shiva notes three essentials to farmers’ agricultural practices: seed sharing, exchange of ideas, and knowledge gained by working with indigenous seeds (2001; 2004; 2012a). She accuses MMB of criminalizing the farmers’ practice of seed saving by manipulating the World Trade Organization’s (WTO) Agreement on Agriculture, via the sections on Intellectual Property Rights (Shiva, 2010a; 2010c; 2013b; Shiva et al. 1999). In Shiva’s opinion, Article 27.3 of the TRIP’s agreement (“[P]arties shall provide for the protection of plant varieties either by patents or by an effective *sui generis* system or by any combination thereof”) was incorporated into the Indian Patent Act to prevent farmers from seed saving and exchange, thereby threatening their livelihood.

In a co-authored article on globalization and seed security, Shiva and colleagues posit that Multi-National Corporations’ monopoly actually enslaved farmers. They regard this advancement of genetic technology to be fundamentally “dictatorial, distorted…coercive” and “anti-people” (Shiva et al. 1999: 611). In recent publications, including “Seed Monopolies, GMOs and Farmer-suicides in India” (2013c), Shiva draws a direct link between farmer-suicide and MMB’s exercise of seed patency. She states:

We have been studying the creation of seed monopolies since 1987, during the period of the Uruguay Round of the GATT, when corporations like Monsanto pushed intellectual property rights over seeds and life forms into TRADE treaties. This led to the TRIP’s agreement of WTO.... Every year more than $200 million flows from the Indian peasants to Monsanto. This is at the heart of the intensification of farmer suicides in the cotton belt of India (Shiva, 2013c).

Notwithstanding her own ruthless assaults on MMB, in an article dated 31 October 2014 and, again, in Navdanya’s open electronic letter addressed to the American president and the Indian prime minister before the former’s visit to India on 26 January 2015, Shiva confuses the issue by stating that there is no seed patency because the Indian patent law excludes seeds from being patented (Shiva, 2014a; Navdanya, 2015). However, towards the end of the electronic letter, she again refers to the patents as an instrument of monopolisation pushing numerous farmers into suicide:

The time has come to stop the use of patents by corporations as a tool to exercise ownership on life, to steal our biological and intellectual heritage, to create seed monopolies and push our farmers to suicide, to claim monopolies on medicine and allow our people to die without access to medicine. Humanity and the Earth must come before short-term profits for a handful of companies.

Cornell University's agrarian political scientist Ronald J. Herring vehemently contests Shiva's allegations of monopolisation. He demonstrates that her accusations actually predate Monsanto's commercial launch of the Bt. seeds in India. Herring points out that at the time Shiva started focusing on Monsanto for seed monopolisation, Monsanto was still grappling with field trials. Furthermore, in Gujarat cultivators were already using Bt. cotton - pirated MMB’s Bt. seeds - developed by Navbharat Seeds (Herring, 2006; 2007a; 2008). His is a bi-faceted argument.

First, Shiva reveals an insufficient knowledge on India's patent laws, which do not allow patency on anybody's seed (Herring, 2006). Similarly, Pune University’s Anitha Ramanna and Michigan State University's Melinda Smale demonstrate that the dominant feature of India's policy on agricultural patency adheres to the principle of common heritage or the promotion of free exchange based on the notion that no one has ownership over the chief food plants of the world, which are our common heritage (Ramanna & Smale, 2004). Karine Peschard points out that India's 2001 *sui generis* plant variety protection bill, Protection of Plant Varieties and Farmers’ Rights (PPV & FR) upholds the farmers’ rights to save, share, exchange and sell seeds except “…branded seeds of a protected variety if they are labelled as such (Peschard, 2014: 1091).” The 2002 amendment of the 1970 Patent Act also excluded seeds from being patented. The proposed 2004 Seed Bill, which forbids farmers’ sale of unregistered seeds, has yet to be passed by the Parliament because of the public outcry against it (Peschard, 2014). Since the MMB seeds, just like the seeds of other firms, are not patent-protected, the rising graph of farmer-suicides in India cannot be ascribed *solely* to MMB’s patent monopoly. Herring opines that MMB benefited not from a monopoly but from an advantaged market position thanks to India’s bio-safety regulations (Herring, 2015). Regulatory authorities generally decline permission for hybrids carrying the illegally introduced Bt. trait (Ramaswamy et al. 2012a, 2012b).

### MMB enforcing seed dependence

Although banned by the government in 1998 and Monsanto promised not to commercialize the Terminator technology, Shiva, time and again, alleges that, allured by the amassment of corporate wealth, Monsanto schemed to introduce the terminator technology. Shiva states in “Monocultures, Monopolies, Myths and the Masculinization of Agriculture” that Delta and Pine Land, acquired by Monsanto, possessed a joint patent with the US Department of Agriculture to manufacture genetically modified seeds designed to render the succeeding generation of seeds sterile thus coercing farmers to purchase new seeds for each and every planting (Shiva, 1999). She made a similar public declaration at the UN Meeting of the Parties to the Convention on Biological Diversity in March 2006 in Curitiba in Brazil (Herring, 2006). Interestingly, Shiva admitted, in a publication co-authored with Kunwar Jalees, that the ‘Terminator technology’ was not commercialized in India and that Monsanto did not employ the technology because of public protests. Nevertheless, she and Navdanya have not ceased making accusations with regard to farmer-suicides (Shiva & Jalees, 2006; Navdanya, 2012). The following excerpt from “Resources, Rights and Regulatory Reform” illustrates her continued accusation (Shiva, 2006: 86; see also Herring, 2010: 616):

Pushed into deepening debt and penury by Monsanto-Mahyco and other genetic engineering multinationals, the introduction of Bt. cotton heralds the death of thousands of farmers (…). These seeds kill biodiversity, farmers and people’s freedom - for example, Monsanto’s Bt. cotton, which has already pushed thousands of Indian farmers into debt, despair, and death. Bt. cotton is based on what has been dubbed ‘Terminator Technology,’ which makes genetically engineered plants produce sterile seeds.

To date, there have been no field trials on this particular technology or its application (Ledford, 2013; Herring, 2006; 2007b). Had Monsanto employed the technology, the Bt. gene would not have been used in the 1990s to hybridize with indigenous cultivars, and Indian farmers would not have replanted the saved “terminator” seeds (Herring, 2007b; 2010). Farmers have replicated the transgenic seeds because, unlike the hybrid seeds produced with two distinct parental lines, these Bt. seeds remained self-pollinating (Shah, 2005; 2008). However, seed-saving remains problematic for the users of the filial-1 hybrid seeds which, though not sterile, produce off-spring with irregular phenotypes, thereby forcing farmers to be market-dependent for each planting (Gutierrez *et al*., 2015). Therefore, it is hybridization, not Terminator technology, that makes Bt. cotton farmers market-dependent (Ramamurthy, 2011). Furthermore, Kranthi and others allude to the fact that yield increase and insect resistance are greatly contingent upon the cultivar into which the soil bacterium Bt. is planted rather than the trait itself, which is solely toxic to bollworms and few other insects (Kranthi, 2012; Stone & Flachs, 2015; Herring & Rao, 2012; Naik *et al*., 2005). Transgenic technology was intended chiefly to counter the onslaught of the American bollworm that accompanied the rapid adoption of hybrid cotton since the 1960s (Shah, 2008). Commenting on this evolution, socio-cultural anthropologist Glenn Davis Stone points out that far from being a mere ecological coincidence, these bollworms were an outcome of rising cotton cultivation, the use of pest-inducing seeds and chemicals, and the proliferation of hybrid seeds (Stone, 2011a; 2011b).

### The destruction of seed options

Biotech opponents ascribe the extensive adoption of Bt. cotton by farmers to being duped by MMB into giving up their indigenous varieties. Farmers gave up their own seeds due to misleading advertisements and mistakenly thinking that their friends or relatives did the same (Shiva 2010a; 2010b; 2010c; Shiva, 2014d; Spinks, 2011). This systematic removal of indigenous varieties, they claim, is largely responsible for farmer-suicides in India. Responding to *The New York Times* journalist Michael Specter, Shiva says (Shiva, 2014d):

If Specter had actually travelled across the cotton belt in Maharashtra State (…) he (…) could have met and spoken to the family of 7 left behind by Ganesh, in Chikni village, following the repeated failure of his Bt. Cotton crop. Ganesh had no option but to buy more (…) and try his luck multiple times because Bt. Cotton was the only cotton seed in the market, brilliantly marketed under multiple brand names through Licensing Arrangements (…) with Indian companies. Multiple packages, multiple promises but the contents of each (…) is the same: it’s all Bt. It’s vulnerable to failure because of too much or too little water, reliant on fertilizer, and susceptible to pests without pesticides, all additional costs. The farmer, with a field too small to impress Specter, does not choose Bt. Cotton of his free will. That choice is dictated by the system Specter attempts to hail.

According to Shiva, the erosion of indigenous seeds and the concomitant dependence on the GM patented variety resulted in deep indebtedness - the most tragic manifestation of which was suicide (Shiva 2012b). The Acharya Report also notes the disappearance of traditional variety seeds and ascribes it to the craze for Bt. seeds (Ministry of Agriculture, 2012). For instance in Andhra Pradesh by 2006 nearly 90% of farmers had switched to Bt. cotton. Consequently, due to a lack of demand for the non-Bt. variety, some merchants stopped stocking it (Herring & Rao, 2012). Dominic Glover (Institute of Development Studies) and Stone convincingly illustrate how MMB's clever marketing strategy was responsible for the *craze* by using the “farmer train farmer” strategy, whereby influential farmers explained to common farmers the new technology. MMB graciously provided free samples and trusted village figures, such as the *sarpanch* (village head), were employed to win the farmers’ confidence and establish credibility. Moreover, MMB even enlisted banks to boost sales by convincing them about the benefits of the transgenic technology and, thus, the benefits of providing credit to the farmers. It even provided a ready line of credit to the banks. Consequently, these banks rushed/pressured farmers to purchase Bt. cotton (Glover, 2007b).

Stone’s analysis demonstrates that it was emulation rather than the farmers’ own appraisal of the seed that influenced their choice (Stone, 2007a; 2007c; 2010a; 2010b; 2011b). Common farmers like to build on other successful farmers’ experiential wisdom. They are open to emulating these farmers, whose seed choices are determined by their inclination to try new seeds each season. It was this emulation that caused the seed *craze* in Warangal district (Stone, 2007a; 2007b). Key to this performance lapse of the technology is, in Stone’s analysis, the’*de-skilling of farmers*.‘In Warangal, for example, he is convinced that the transgenic technology was introduced into a system wherein a widening disjuncture between farmers‘knowledge, environmental learning, and their decision making process was already underway. He demonstrates that the farmers‘natural learning curve was impeded by their unfamiliarity with the technology, its inconsistent temporal effects, and the rapid-paced introduction of novel GM varieties. Stone lists some other factors responsible for the *de-skilling* process: the same seeds being sold under numerous brand names, rapidly changing seed products, contradictory and confusing claims by both proponents and opponents of the transgenic cotton, the ever-present identically labeled spurious seeds, highly variable seed and product features, the ubiquity of advertisements believed to be factual, the unpredictability of the pest population and expression of the transgenic protein, the presence of spurious and adulterated pesticides, transgenic cotton‘s seasonal and intra-brand performance variations, unreliable information from the mass media, the presence of numerous actor-spokesmen, such as NGOs and activists, influencing the farmers‘decision-making process, cultivation information from unreliable sources and the lack of proper agricultural extension services etc. (Stone, 2004; 2007a; 2007c; 2010a; 2010b; 2011b). Some of the best examples of this *de-skilling* were the seed selection fad and the pesticide treadmill in Warangal (discussed below) with some farmers over-spraying. Based on these observations Stone concludes that the fundamental problem with the Bt. cotton cultivation in India lies with the fact that the Indian cultivators are expending money on something for which they have insufficient knowledge (Stone, 2015).

Stone points to the success experienced by the Gujarati farmers with Bt. cotton cultivation to contrast the *de-skilling* impact in Warangal. He discovered three pertinent factors. First, Gujarati farmers were using indigenously adjusted seeds often produced by the farmers themselves with the Bt. parent line secured from the banned Navbharat-151 seeds ubiquitous in Gujarat from 1999 to 2001. Second, they experimented on smaller plots with loose seeds available in the market instead of the one-acre plots in Warangal. Third, some Gujarati farmers still cultivated non-hybrid cotton (Stone, 2007b). Maastricht University‘s social scientist Esha Shah ascribes the success of Gujarat‘s affluent Patel farmers to two other significant factors viz. the similarities of the transgenic technology with the Green Revolution technology with which the Gujarati farmers had experienced success and the cheap, young, female, and child labour force from Rajasthan at their disposal (Shah, 2005; 2007; 2008). Thus, neither the transgenic technology in itself nor the transgenic seeds are sole factors responsible for Bt. cotton‘s underperformance. The impact of other factors together with higher prices farmers must pay for the transgenic seeds can put farmers in a more precarious position than their non-Bt. counterparts (Glover, 2010a; 2014; Dowd-Uribe et al. 2014).

### A perfect recipe: Indebtedness or suicide?

Opponents of biotech argue that the increased costs for agro-inputs such as seed, water and chemical inputs contribute greatly to the financial burden of farmers. The following excerpt quintessentially sums up their contention: “As Monsanto's profits grow, farmers’ debt grows” (Shiva, 2013d). Rising production costs of cotton, once regarded as lucrative, have forced farmers to sell their assets, even their wives' *mangal sutra* or a portion of their land to pay their debts (Shiva and Jalees, 2006). The Acharya Report observed that Bt. cultivation required nearly ₹48000-54000/acre, chiefly because of the expense incurred on inputs vs. ₹8000-12000 required by non-Bt. (Ministry of Agriculture, 2011). Opponents claim that MMB’s seeds were initially available for free or at minimal price. The corporation's patent monopolisation caused the exorbitant hike in price from ₹7-9/1 kg seed in 1991, i.e., before the introduction of Bt. cotton, to ₹350 in 2003, and between ₹1650 and ₹1850 in 2004 for 450 g (Shiva, 2010c). After 2004, with the intervention of government, MMB reduced the prices to ₹1,600/450 g. packet, and still earned ₹34 billion (Navdanya, 2012). Others, like Sainath and the Acharya Report, also blame the price rise after the introduction of MMB’s Bt. cotton for the individual farmer’s unsustainable debt, a reason for the majority of suicides (Sainath, 2009, 2009; Ministry of Agriculture, 2011). Shiva concludes that pushing seed costs beyond market-regulated prices could be considered as new colonialism, even worse than the speculation- and tax-driven Bengal famine of 1943 (Shiva, 2011a; 2011c; 2013b; 2014d; 2014e; Shiva & Jalees, 2006). In the aforementioned open letter to the Indian prime minister and the American president Obama, Shiva admits that while the exorbitant price of the MMB cotton is not the result of patents, because it was declared illegal, the company “goes outside the law to collect royalties as ‘technology fees’” (Navdanya, 2015).

Second, the biotech opponents note that the increased need for chemical applications ups production costs. More than 50% of the agrochemicals used in farming is for cotton cultivation to control bollworm and this is only 5% of the total cropped area. A cotton farmer spent in 2005 ₹1100 for agrochemicals (Shiva & Jalees, 2006). Devinder Sharma argues that transgenic pest-resistance contributed paradoxically to an increased pesticide use for Bt. Cotton (Acres USA, 2004). That was not always the case. In 2003 Shiva stated that the transgenic cotton required less pesticide than its local counterpart (Shiva & Jafri, 2003), but the situation changed. The newly created paradox could be explained by the practice of monoculture, increased chemical fertilization, rising resistance of bollworms, new pests, and the destruction of predator species that control pests, as factors responsible for pest-proneness in Bt. cotton (Shiva, 2010c; Ho, 2011). These factors explain the 13-fold hike in pesticide use in comparison to the pre-Bt. cotton era (Shiva, 2010b; 2010c). While MMB’s Bollgard cotton seeds were advertised as resisting bollworms, discouraging farmers from using pesticides, the opposite seems to be true because bollworms have now become Bt. Resistant (Shiva, 2015).

Third, Bt. cotton requires significantly more water. To meet irrigation expenses many cultivators resort to borrowing from money sharks at exorbitant interest rates. Heavy capital investment and interest rates force farmers to try to produce more in order to cover their debt; however, with crop failure, the possibility of suicide looms (Shiva & Jalees, 2006; Shiva & Jafri, 2003). A study by the Center for Human Justice and Global Justice (CHRJ) confirms that Bt. cotton seeds require much more water than non-Bt. seeds (Center for Human Justice and Global Justice, 2011). However, the fact remains that cotton, be it transgenic or not, is water-intensive and susceptible to failure when inadequately irrigated. Herring mentions the 2004 crop failure due to drought. The well-irrigated transgenic crop yielded a better harvest than similar well-irrigated non-transgenic crop (Herring, 2008; Herring & Rao, 2012). Stone points out that those earlier varieties of MMB's GM cotton did require more water than subsequent varieties (Stone, 2011a). Obviously, for a subsistence farmer in an irrigated area who lacks farm management skills and spends a hefty sum on seeds and pesticides, the depleting water tables and irrigation needs are an added burden (Gutierrez *et al*., 2015).

A study of the Andhra Pradesh Coalition in Defence of Diversity, a consortium of civil society organizations, came to the same results. They observed that Bt. farmers spent nearly 3 times more on seed costs and significantly more on fertilizers and pesticides. The average expense for 3 years on seeds and pesticides/acre for Bt was ₹1557 and ₹2571 respectively for MMB Bt cotton vs ₹2571 and ₹2776 for non-Bt farmers. The average total production cost was ₹11,594 for Bt and ₹10,556 for non-Bt (Qayum & Sakkhari, 2005).

A similar observation is made by Guillaume Gruère, Purvi Mehta-Bhatt and Debdatta Sengupta who presume a connection between exorbitant technological costs, unfavourable yields, and farmers' indebtedness as resulting in suicide (Gruère et al. 2008; Gruère & Sengupta, 2011). Before the reduction of the seed costs, following the intervention of the Andhra Pradesh government in 2006, transgenic seeds cost almost five times more than non-transgenic (for instance, ₹300 for the non-Bt. DCH32 vs. ₹1650 for the transgenic seeds of the 450 g packet) (Gruère et al. 2008). Although there was little difference in the production costs of Bt. and non-Bt. hybrid cotton, costs for GM cotton were exorbitant because the firm was the first and sole provider of the transgenic genes in India and imposed trait fees ($30 of the $45 charged for a 450 g of Bt. cotton seed)- as much as 1/3 of the retail price (Basu & Qaim, 2007; Arora & Bansal, 2012; Lalitha, 2004; Lalitha et al. 2008; Murugkar et al. 2007; Ramaswamy et al. 2012a, 2012b; Rao, 2008). Other firms failed to provide viable competition; as a result, Monsanto exerted considerable sway on the pricing mechanism. Illegal seeds, correspondingly, cost almost 2/3 of the legal Bt. seeds (Lalitha et al. 2008; Basu & Qaim, 2007). With the rapid adoption of transgenic cotton by farmers, seed firms frantically attempted to collaborate with MMB (Ramaswamy et al. 2012a, 2012b). Devparna Roy notes that about 50 firms have made licensing agreements for the Bt. gene with the MMB (Roy, 2015). Even with price reductions, the transgenic cotton, at $19 for a 450 g packet, is still relatively expensive given that households with ₹300 ($5) or more are considered indebted and ₹25 ($0.40) is considered a sufficient daily income for rural households (Kennedy & King, 2014; National Sample Survey Organisation, Ministry of Statistics and Programme Implementation, 2005). However, this fact needs to be evaluated against the profitability of Bt. cotton cultivation.

Countering accusations by Bt. critics, Herring and Stone have shown that transgenic cotton actually reduced pesticide use. Referring to O.M. Bambawale et al.’s comparative study on integrated pest management and pesticide use in isogenic Bt. and non-Bt. MECH 162 seed and conventional hybrid seed, Herring shows that Bt. had less bollworm attack, thus needing less pesticide application (Herring, 2006; Bambawale *et al*., 2004). Stone’s panel study, from 2003 to 2007, showed that the introduction of the transgenic cotton in Warangal, resulted in 54.7% reduced pesticide usage (2011b). Some studies warn about the rising presence of secondary pests resulting eventually in more need for pesticide application in the future (Gutierrez *et al*., 2015). In Maharashtra and Gujarat, 45% & 76% of pesticide application, respectively, was used to counter sucking pests vs. 24% & 7% against bollworms (Stone, 2011b). At all-India level, the adoption of Bt. cotton has not proven to be a boon with regard to pesticides, as their use after steadily declining to 4623 metric tonnes in 2006, increased again to 11598 metric tonnes in 2013, surpassing even the 2000 level of 10988 metric tonnes (Gutierrez *et al*., 2015; Kranthi, 2014; Nagrare, 2009). The absence of farmers' management skills, as Stone noted, may be responsible for the alleged hike in pesticide application. Stone characterizes cotton as “the classic pesticide treadmill crop” (Stone Glenn Davis. Biotechnology and Suicide in India. Anthropology News 2002a: 5). He identifies it with the disproportional application of pesticides on the New World Hirsutum hybrids in Gujarat, Maharashtra and Andhra Pradesh in the 1990s (Stone, 2011b).

### Economic violability *in extremis*?

Biotech opponents contend that there is a *necessary* connection between the failure of Bt. cotton to live up to its yield-return expectations and cotton farmers’ suicide. For instance, the RFSTE study on Bt. cotton yield in Nimad (MP) and Vidharbha attributed crop failure and suicides to agronomic factors - leaf curl, reddening, etc. There have been repeated crop failures since the illegal trials in 1998 and Bt.’s subsequent commercialization in 2002. While many farmers lost their entire crop, others yielded on average 3 quintals/acre at an average expense of ₹6000/acre (Shiva & Jalees, 2006; Shiva, 2010b; 2010c; 2014d). T.V. Jagadisan, former managing director of Monsanto India, also believes crop failures precipitated the suicides of many (2010). Some news reports supported this theory (rediff Business rediff.com, 2010). Second, Shiva et al. compared the failure of the Bt. cotton vs. organic cotton. Farmers using organic seed fared better than their Bt. counterparts all round, in terms of yield, pesticide use, and expenditures. Contrary to Monsanto's projection of a 50% increase in yield for Bollgard cotton, various Navdanya studies revealed that organic and non-Bt. varieties produced more than Bt. Cotton, and that non-Bt. farmers’ net income surpassed that of the Bt. farmers. Shiva references the RFSTE study showing the net income of the organic/Bt. farmers was ₹8000 and 4168.13/acre in 2011 (Shiva, 2010c). Madhya Pradesh cultivators of Bt. Cotton, on the other hand, lost ₹543/acre while cultivators of the non-Bt. variety gained a profit of ₹6315 per acre. In Karnataka the former lost ₹1285 per acre, the latter gained ₹3750/acre (Shiva & Jalees, 2006). In Andhra Pradesh Bt. yielded 12 times less cotton, used 4 times less pesticide, and profits were 100 times less than Monsanto's claim (Shiva & Jalees, 2006). Also another study, the Sakkhari-Qayum study, observed that non-Bt. outperformed Bt. by yielding ₹4787/acre against ₹2032/acre, 708 kg/acre against 649 kg/acre (Qayum & Sakkhari, 2005). These facts helped Shiva and Jalees establish the idea that transgenic cotton is, indeed, a suicide/genocide/homicide-inducing technology (Shiva & Jalees, 2006, 43f):

High costs of cultivation, and low returns have trapped Indian peasants in a debt trap, from which they are escaping by taking their lives. More than 40,000 farmer-suicides have taken place over the past decade in India. However, these are not suicides – this is homicide, it is genocide. More than 90% of farmers who died in Andhra Pradesh and Vidharbha in the 2005 cotton season had planted Bt. Cotton. Genetic Engineering is killing Indian farmers (Herring & Gold 2005).

Shiva concludes that in globalization, corporations gain and small farmers bear the risks. Therefore, she challenges Monsanto’s claim of improving farmers’ lives (Shiva, 2009). Studies confirming farmer-suicide as a result of the failure of the transgenic technology are frequently unsatisfactory for two reasons. Firstly, they ignore the thriving counterfeit seed market, which might have contributed to crop loss (Gruère & Sengupta, 2011). The unbridled spread of spurious seed often made it difficult to distinguish between non-Bt. seeds and Bt. (Herring, 2006; 2007a; Gruère & Sengupta, 2011). Herring asserts that counterfeits are undoubtedly responsible for the scepticism associated with the results of Bt. seeds (Herring, 2008). This confusion, resulting from approximately 165 Bt. varieties in 2007, actually helped the spread of the use of spurious seeds (Gruère & Sengupta, 2011). Kranthi ascribes up to 28% of the fake cotton seeds do not contain the beneficial transgene (Herring, 2008). A Central Institute for Cotton Research (CICR) study reported that 46% of seeds tested were adulterated. These analyses show that around 30% of the seeds available in Vidarbha were fake (Herring, 2008; Herring & Rao, 2012; Herring & Kandlikar, 2009; Padma, 2006). In Andhra Pradesh, available seeds were not certified because certification was voluntary. In the absence of certification and control, counterfeit or duplicate seeds without the Bt. transgene, are susceptible to bollworm infestation; farmers who innocently buy them face hurdles. Counterfeits may be of the F2 variety with an assorted lineage (Herring, 2007a; 2008; Gruère & Sengupta, 2008). The huge gap between demand and supply of Bt. seeds, the better performance of some unofficial Bt. seeds, the absence of regulation, and the cheap price of the unofficial varieties etc. abetted the sporadic presence of duplicates in the market (Herring 2008). Stealth seeds made analysis of cotton’s performance more difficult since no account of acreage cultivated exists (Herring, 2007a; 2013; Herring & Kandlikar, 2009).

Secondly, the transgenic trait is not intrinsically yield-enhancing. However, any claims of the inevitable failure of the transgenic cotton needs to be taken with the proverbial grain of salt. Cued by Bambawale et al.’s study, Herring shows that the transgenic cotton yielded 710 kg/ha and an income of ₹10,507/ha. He also points to the 2012 Kathage and Qaim’s study that showed an increase of 24% in yield, 50% in profits, and 18% improvement in living standards (Herring, 2006; 2013; Bambawale *et al*., 2004; Kathage & Qaim, 2012). Stone's panel study also estimated an 18% average crop yield increase (Stone, 2011b). However, these benefits should not be exaggerated either, for on the macro level, total adoption has seen a decline in yield. The rising trend in suicide-prone Maharashtra and Andhra Pradesh began when GM adoption was quite meagre; now the yield has declined to the levels prevailing before the total adoption (Stone, 2012a; 2012b).

## Is there a connection between monsanto’s bt. Seed and farmer-suicide?

Scepticism over the appropriateness of Bt. cotton for India’s farmers notwithstanding, Shiva’s establishment of a *direct* link between MMB’s seed monopolisation and farmer-suicides remains a towering assertion. Nevertheless, history and inter-state data do not support it. Firstly, as Gruère et al. noted there are no clear statistics of which suicides were due to the cultivation of transgenic cotton (Gruère et al., 2008).

Secondly, Srijit Mishra’s study on this topic, between 1995 and 2001, shows farmer-suicide rates in Andhra Pradesh, Gujarat, Haryana, Punjab, Tamilnadu, Karnataka and Rajasthan to be unstable, while increasing in Maharashtra and Madhya Pradesh. His study shows that the suicide rate for male farmers increased from 1995 to 2001, i.e., before the Bt. introduction. Male suicides in Andhra Pradesh, Karnataka, Madhya Pradesh, Maharashtra and Tamilnadu had already been quite high (Mishra, 2006a).

Thirdly, as Stone argues, the peaks in farmer-suicides happened either before farmers adopted the transgenic cotton or when there was limited adoption. Stone points out that in 1998, a peak year in Andhra Pradesh, the transgenic cotton had not been introduced and 2002, another peak, marked the official introduction of the transgenic cotton. These two peaks confirm that there is no *causal* connection between Monsanto's seed monopolisation and farmers’ suicides. Stone estimates that, between 2002 and 2006, transgenic cotton held a meagre 0.4-5.6% of the national total for cotton cultivation. As discussed above, in the Warangal district of erstwhile Andhra Pradesh, yields increased after the introduction of the transgenic cotton (Stone, 2012a). Ian Plewis’ calculation of the suicide rate from 1996 to 2011 in the nine cotton producing states shows a constant suicide rate in Madhya Pradesh, Karnataka and Tamilnadu, a varying rate in Rajasthan, a steady rise in Andhra Pradesh and Haryana, a reduction in Gujarat until 2004 with an increase in Punjab and an increase in Maharashtra until 2005 followed by a reduction. The suicide rate for men and women in all these states showed a rise until 2005 and a subsequent decline thereafter; for women, the decline was steady. In short, there is no evidence to support a necessary correlation between Bt cultivation and farmer-suicide. Maharashtra and Punjab, respectively, have recorded both increases and declines since adopting Bt. Economic factors, therefore, likely influenced the suicide rate (Plewis, 2014). Fourthly, when conditions are favourable, GM cotton has proven to be economically viable; e.g., in Gujarat the GM cotton contributed up to a 24% yield and 50% increase in income for GM farmers (Herring, 2013).

Fifthly, the non-cotton cultivating state Kerala is also infamous for a high number of farmer-suicides (Sadanandan, 2014; Münster, 2012). This demonstrates there is no *necessary* link between the cultivation of GM cotton and farmer-suicide.

Sixthly, other studies point to the fact that farmer-suicides took place in India before the 1990s. Sucha S. Gill, for instance, noted the occurrence of farmer-suicides in Punjab since the mid-1980s (Gill, 2005). Ergo, contrary to Shiva’s assertions, farmer-suicides were not unheard of prior to the Mahyco-Monsanto alliance. Hence MMB’s seed monopolisation is not the “*smoking gun”*. Nevertheless, a thoroughgoing ethnographic study is still required to unearth the root(s) of this epidemic.

There is a definite link between economic factors and farmer-suicide, according to Gutierrez et al. They demonstrated that farm size and yield - the yardsticks of poverty and risk - as well as the acreage cultivated, and persistent insecticide application, are the true culprits. They observed that suicides declined with an increase in farm size, cultivated acreage and yield. They concluded that poor farmers, particularly in places with poor but highly fluctuating yields, find it harder to meet the expenses of Bt. cotton farming and, thereby, incur onerous debt and likely suicide (Gutierrez *et al*., 2015). As pointed out by Ainahalli R Vasavi, debt has been a characteristic feature of Indian farming. The situation has been exacerbated for marginal agriculturists wanting to finance new technology by the decline of formal credit sources and interest rates of 5–10% (Vasavi, 2012). It can be argued that there is a connection between the high seed costs, the need to purchase seeds each planting season, poor returns and suicide by indebted farmers. Glover notes that the high costs of transgenic cotton can have a two-pronged impact on farmers. On the one hand, the Bt. toxin, which guarantees a protection against insects, does not ensure an enhanced yield; especially, in seasons when there is no serious pest pressure. Farmers pay a higher price for seed without being recompensed by a greater yield. On the other hand, the Bt. toxin offers no protection against secondary pests or natural calamities (Glover, 2010b). A similar situation can be noted in the non-cotton growing state Kerala which has recorded a high suicide rate. There the cultivators of crops like vanilla, pepper, rubber, coffee, banana and ginger incur unsustainable debt owing to an unfavourable expenditure-return ratio arising from high investment, crop loss and price fall (Vasavi, 2014). Vasavi notes that farmers grappling with debt face intense apprehension. The lives of the distressed farmers revolve around dealing with creditors. In a major portion of rural India, where gendered roles still exert a profound effect, the failure of a man to fulfill familial and individual obligations, worsens his life (Vasavi, 2014; 2009; 1999). This assessment is shared by Priti Ramamurthy. Her ethnographic research on gendered ideologies and labour relations among cotton producers in rural Andhra Pradesh construes farmer-suicide as symptomatic of a masculine identity crisis imbued into all males by the society from childhood. A large portion of male identity is derived from his role as family’s bread-winner. By the late 1990s, the decline in crop yield, the rise in production expenditures, and the low financial return for their crop made life hard for the farmers. This societal delineation of the masculine identity finds its cruelest manifestation in suicide by those unable to meet those expectations (Ramamurthy, 2004; 2010).

## What is the relevance of critics’ claims abouttransgenic technology?

Many past peer-reviewed studies dismissed the analyses by biotechnology opponents as lacking transparency; however, their assertions deserve to be heard and evaluated in light of the deterioration of India’s agrarian community following the country’s economic liberalization. The underlying thesis of the globalization critics and the biotechnology opponents is the same: India’s agrarian crisis and farmer-suicides are revealing the political and economic marginalization of the agrarian sector (Vasavi, 2009).

In the wake of India’s economic liberalization, many critics of mainstream economics viewed farmer-suicides as repercussions of dwindling protective factors such as subsidies and credit availability (Ramaswamy, 2002; Ramani & Thutupalli, 2015) or by increased cultivation costs (Patnaik, 2003). Some studies suggested that the lifting of trade barriers exposed the Indian farmers, especially cultivators of export-oriented cash crops, to the vulnerability of global trade competition (Nagaraj, 2008). Jonathan Kennedy and Lawrence King's observation that there exists a strong correlation between cash-crop cultivation, marginal land-holdings, indebtedness, and the rate of farmer-suicides, assumes greater significance (Kennedy & King, 2014). It has also been observed that the production costs incurred by farmers skyrocketed in the reform period. The increased production cost is attributed to the reduction of government investment (Merriott, 2016). Various studies attest to this reduction (Dandekar et al., 2005; Mishra, 2006b; The National Bank for Agriculture and Rural Development, 2015). This lackadaisical investment reveals itself vis-à-vis irrigation. Anoop Sadanandan’s study shows that 1/3 of agricultural land is irrigated, illustrating the excessive dependence of Indian farmers on rainfall (Sadanandan, 2014). N. M. Kale’s study showed that only 15% of the arable land in selected districts of the suicide-prone Vidarbha were irrigated, which adversely affected cultivation and yields (Kale, 2011). Furthermore, Kale and co. have shown that more than 2/3 of the farmers from Vidarbha had no sources of irrigation other than the monsoon rains (Kale *et al*., 2014). Surya Prakash Rao Gedela et al.’s study on suicide of Andhra Pradesh farmers has evinced that the farmers who committed suicide had a lower portion of their land irrigated than those who did not (Gedela *et al*., 2008). Two factors are noteworthy. First, there is a perception that farmers’ needs were overlooked in the execution of neoliberal policies (Randeria, 2007). If the government wanted the progress for agriculturists and rural areas, it would have refrained executing neoliberal policies that wreaked havoc on farmers (Randeria, 2007). Shalini Randeria noted India’s cunning game with its *partial execution* of the reform policies: partial privatization of public sector institutions, partial compliance of rupee devaluation, non-compliance with the privatization of the labour market, and the total compliance with the removal of unprofitable agricultural subsidies (Randeria, 2003; 2007). She argues that the compliance-issues with the aid/loan conditionality do not necessarily affect the granting of future financial supports. She illustrates it with the World Bank-funded Sardar Sarovar project on the Narmada River to construct several dams and a 1200 watt powerhouse. The project was abandoned following protests from a group of national and transnational American and European NGOs who feared the forceful eviction of nearly 200 thousand people - mostly tribal - and the devastation of their sustenance source. However, despite the forced termination of this World Bank project, the absence of supervision by the Bank and the failure of the Government to furnish a rehabilitation policy, the Bank still financed developmental projects requiring the forced eviction of people. In this same vein Randeria contends that the removal of the Quantitative Restriction (QR) on 229 agricultural products in March 2000 was not required by the WTO agreement. Had the Government argued against the removal of these QRs, on the basis of its prospective harmful effects on the poor farmers, they could still have been retained (Randeria, 2007).

Secondly, successive governments have failed to comprehensively appraise and sufficiently respond to the constant suicides. Many reports and policies submitted by scientific committees, such as the National Agricultural Policy for the Enhancement of Agricultural Growth and Village Economy, have seldom been acted upon by successive governments. At the same time some initiatives, which have public representatives and experts as collaborators with the intention of the amalgamation of agriculture and land into neo-liberal economy, have been executed without proper popular consultation, debate and scrutiny. The Indo-US Knowledge Initiative, aiming at the development of fresh technologies like the Biotechnology for the advancement of agriculture in US and India, is an example of this. Its Board consists of the representatives of transnational corporations, with few representatives from the Indian government and agricultural universities. This initiative gives sufficient scope for multinational corporations, like Monsanto and Cargil, to strengthen their position in India. The absence of representation from elected representatives, farmers’ or civil society associations illustrate the evident breach of democratic procedures on issues of agricultural progress -failing to uphold the concerns of the marginal and poor farmers. So, instead of focusing on matters significant to farmers like resource reallocation, strategies to ensure a sufficient price for their products, and crisis intervention, governments accorded increasing emphasis to India’s industrialization, thus aggravating the marginalization of the India’s farmers (Vasavi, 2009). Noteworthy in this context is the fact that India introduced economic liberalization when its switch toward capital-intensive hybridized crops had already been proving to be economically devastating to farmers (Spielman *et al*., 2013). Since the 1970s, such capital intensive agriculture meant the replacement of traditional agricultural practices, i.e., the usage of manure and farmer-saved seeds with hybrid seeds, chemical fertilizers and pesticides (Dutta, 2012). The use of hybrid seeds instead of the traditional variety of farmer-saved seeds made farmers market-dependent, as hybrid seed vigour declines from the second generation onwards (Murugkar *et al*., 2006; Spielman *et al*., 2013). The requirement to purchase hybrid seed each planting season caused a serious economic burden to subsistence farmers for whom seed-saving was a survival strategy (Spielman *et al*., 2013). Pray and Natrajan show that farmers' use of inputs, such as fertilizers, more than doubled each decade since 1971. Fertilizer use which was 16.5 kg/ha in 1971 increased to 69.84 kg/ha by 1991 (Pray & Natrajan, 2012). Hybridization also tends to allow a few firms to control the market, thus providing opportunities for monopolistic pricing (Dutta, 2012). Furthermore, hybridization had two drawbacks: first, the obtainment of a high crop yield requires the appropriate application of inputs together with proper irrigation (Spielman *et al*., 2013) and, second, the constant and exorbitant use of fertilizers and pesticides results in soil depletion and pesticide resistance which, in turn, trigger increased usage of these chemical inputs. In the long run, such an agricultural system necessarily falls short of economic and ecological viability (Ramani & Thutupalli, 2015).

Moreover, India’s governments viewed the crisis as symbolic of the farmers’ personal frailty and dependence. An example is the Karnataka government’s invocation of the recommendations of the Expert Committee on Suicide to instill self-respect and self-reliance among agriculturists. The Maharashtra government has sought the assistance of spiritual leaders like Amritanandamayi to establish spiritual guidance and rehabilitation centers in the suicide-prone Vidarbha region in Maharashtra (Vasavi, 2009). After several suicides in January 2011 in Madhya Pradesh, the state’s agriculture minister downplayed the distress as being a consequence of the farmers’ past shortcomings of destroying land fertility via excessive chemical application (Vasavi, 2012). Consequently, victims rather than structural causes are blamed for the farmers’ predicament (Vasavi, 2009; 2012). Although the national government has initiated some relief measures, such as loan wavers and relief packages for bereaved families of farmer-suicides, it has been criticized as political strategies to win votes rather than reducing agrarian misery. The continuation of farmer-suicides testifies that the root cause of the problem viz. the consistent neglect of farmers and farming in the neoliberal phase, has remained unaddressed (Vasavi, 2012).

In such a scenario, anti-globalization activists, such as Shiva and colleagues, serve as whistle-blowers intending to seize the attention of those considered to have the potential to impact the future of agriculture (Chateauraynaud & Torny 2005; Chateauraynaud et al. 2009). Their contention is clear: the advancement of Bt. cotton is not in the best interest of the public’s welfare. Adopting GM crops will result in the virtual enslavement of subsistence farmers by multinational firms (Barsanian, 2002; Shiva 1989).

## Conclusions

Summing up, the chief components of the allegations by the biotech opponents on the link between MMB’s seed monopolisation and farmer-suicide are: the firm’s thwarting of seed-saving and exchange via patency and use of terminator technology; the clever destruction of farmers’ seed variety; the increased production cost; the insufficient crop-yield and financial returns. Our analysis demonstrates that some components of the biotech opponents’ accusations viz. patent imposition and usage of terminator technology are baseless. However, seed-saving does remain a problem for users of the filial hybrid 1 seeds since these seeds produce offspring with variable phenotypes. We also noted the firm’s monopolistic practices forced Bt. farmers to invest heavily in something for which they lacked the proper management skills. Although the transgenic trait is supposed to contribute to savings on pesticide application, it has not necessarily been so because of farmers’ over-usage and secondary pests. Irrigation presents added problem for those farmers who must invest heavily in seeds and pesticides. While the viability of the Bt. cotton is not disputed, the national and inter-state data do not support transgenic cotton as an overall boon to the Indian farmers. The equation is simple: a Bt. farmer facing an unfavourable expenditure-yield ratio can find himself in a more precarious position than a non-Bt. farmer. The scepticism over the appropriateness of the Bt. cotton notwithstanding, Shiva’s *direct* link between MMB’s seed monopolisation and farmer-suicide stand tall. Still, history and inter-state data do not support it. However, there is a definite association between economic factors associated with Bt. cotton farming and farmer-suicide. Furthermore, the overall thesis of the biotech critics needs to be re-evaluated within the context of India's economic liberalization and globalization. The prevailing view is that farmers' needs and concerns were disregarded by successive governments. The fact remains that, by the time India adopted Bt. cotton, hybrid cultivation had already proven detrimental and provided fodder to the critics. Also, despite the high number of farmer-suicides, India’s governments failed to sufficiently address the crisis, attributing these suicides to personal weaknesses vs. systemic problems. In this scenario, via alarms, public protests, lawsuits, and radical media messages, pro-farmer activists act as whistle-blowers attempting to seize the attention of those in power. Their continued advocacy is significant in the context of a weak democracy like India, where market forces are given precedence over farmers’ welfare, at least until the next election (Scoones, 2005; 2008; Vasavi, 2009).

## Abbreviations

CHRJ: Center for Human Justice and Global Justice
DBT: Department of Biotechnology
DOE: Department of Environment, Forest and Wild Life
GEAC: Genetic Engineering Approval Committee
ICAR: Indian Council of Agricultural Research
KRRS: *Karnataka Rajya Ryota Sangha*
MMB: Mahyco-Monsanto Biotech
PIL: Public interest litigations
PPV & FR: Protection of Plant Varieties and Farmers’ Rights
QR: Quantitative Restriction
RAFI: Rural Advancement Foundation International
RCGM: Review Committee on Genetic Manipulation
RFSTE: Research Foundation for Science and Technology
USDA: United States Department of Agriculture
WTO: World Trade Organization
